# Oral granulated Chinese herbal medicine (YXBCM01) plus topical calcipotriol for psoriasis vulgaris: study protocol for a double-blind, randomized placebo controlled trial

**DOI:** 10.1186/1745-6215-15-495

**Published:** 2014-12-19

**Authors:** Shefton Parker, Anthony Lin Zhang, Claire Shuiqing Zhang, Greg Goodman, Zehuai Wen, Chuanjian Lu, Charlie Changlie Xue

**Affiliations:** School of Health Sciences, RMIT University, PO Box 71, Bundoora, Victoria 3083 Australia; The Dermatological Institute of Victoria, 8-10 Howitt Street, South Yarra, Victoria 3141 Australia; Guangdong Provincial Academy of Chinese Medical Sciences & Guangdong Provincial Hospital of Chinese Medicine, Guangzhou, 510120 China

**Keywords:** calcipotriol, Chinese herbal medicine, PASI, psoriasis, Psoriasis Area Severity Index, randomized controlled trial, YXBCM01

## Abstract

**Background:**

Probably related to immune dysfunction, psoriasis vulgaris is a chronic, painful, disfiguring and disabling dermatological disease, carrying an increased risk of serious comorbidities. Current conventional therapies can be costly, show risks of side effects and have limited efficacy, with relapse common on treatment cessation. Chinese herbal medicine is effective in treating psoriasis vulgaris. However, any benefit of adding Chinese herbal medicine to conventional treatments when treating psoriasis vulgaris is yet to be determined.

**Methods/design:**

This is a pilot randomized, placebo controlled, double-blinded trial. The pilot is primarily to determine the feasibility of undertaking a full size randomized trial. Thirty participants with psoriasis vulgaris and Psoriasis Area Severity Index (PASI) scores ≥7 and ≤12 will be included. Participants will be randomized (in a 1:1 ratio) to receive oral granulated Chinese herbal medicine YXBCM01 plus topical calcipotriol 0.005% or oral YXBCM01 placebo plus topical calcipotriol 0.005% treatment for 12 weeks, with a 12-week follow-up phase. The Chinese herbal medicine or placebo will be administered orally as dissolvable granules. The primary outcome measure will be PASI change (%) from baseline to the end of treatment phase. Secondary outcomes will include safety, key psoriasis-related cytokine changes (for example, IL12, IL17 and IL 23) during the entire trial and symptom relapse rates at the end of the follow-up phase.

**Discussion:**

The study will evaluate the feasibility of a randomized controlled trial investigating combined conventional and Chinese herbal medicine therapy for psoriasis vulgaris. The ingredients of YXBCM01 were selected based on literature, the expert opinion on herbal medicine and pre-clinical evidence, for instance Chinese herbal medicine possesses anti-inflammatory or antiproliferative properties.

**Trial registration:**

Australian New Zealand Clinical Trials Registry ACTRN12614000493640.

## Background

Psoriasis is described by the World Health Organization as a ‘chronic, non-communicable, painful, disfiguring and disabling disease for which there is no cure’ [1]. The pathogenesis of psoriasis is understood to be related to immune system dysfunction, with increased function and infiltration of immune cells such as lymphocytes and hyperproliferation of keratinocytes, with consequent inflammatory changes [2]. Psoriasis vulgaris (or plaque type psoriasis) is the most common type of psoriasis, making up 90% of psoriatic cases [3]. Characterized by well-defined, raised red patches (plaques) with adherent thick silvery scales, psoriasis vulgaris is a relatively common dermatological condition [3]. Psoriasis has the highest prevalence of any autoimmune diseases in the USA (between 3.1 and 5.1%) [4, 5]. Related healthcare costs are estimated at US $11.3 billion per annum, with further losses in productivity calculated at around $16.5 billion per annum [6, 7]. An Australian psoriasis prevalence study calculated an even greater percentage with an estimated 6.6% of the adult population affected [8].

Increased comorbidity risks linked to psoriasis include cardiovascular disease, diabetes, cancer, hypertension, obesity, depression and osteoporosis. Death from cardiovascular disease alone has been shown to be increased by 57% in patients with psoriasis [9, 10]. On average, people with severe psoriasis die 5 years younger than those without psoriasis [11]. Treatment guidelines recommend monitoring plaque severity, most commonly utilizing the Psoriasis Area Severity Index (PASI) [12]. In addition, quality-of-life estimation using a validated instrument, such as the Dermatology Life Quality Index, is also recommended [13, 14].

First-line therapies typically involve application of topical drugs (such as corticosteroids and vitamin D analogues). Second-line therapies consist commonly of ultraviolet light therapy (UVB or UVA), classical systemic drugs, such as methotrexate, retinoids and cyclosporine, or, more recently, biologic drugs [15–17]. Systemic biologics, although often effective, are used cautiously, owing to concerns surrounding the safety profile for their long-term use [6, 18]. Associated costs of biologics can also be relatively high [19].

Chinese herbal medicine has been used frequently for symptoms associated with psoriasis. The use of Chinese herbal medicine can be dated back to approximately 5000 years ago [20]. Clinically, to treat disease, Chinese herbal medicines are combined into formulae for topical or oral administration. Administered alone, or alongside conventional therapies, both forms of Chinese herbal medicine are clinically utilized to treat psoriasis vulgaris [21]. Recently published clinical guidelines for Chinese medicine recommend several different topical and oral formulae, to be used for psoriasis. Published research evidence evaluating the use of these medicines in psoriasis is, however, lacking [22, 23]. Previous clinical trial evidence indicates, when combining oral Chinese herbal medicine with pharmaceutical drugs, that there may be add-on effects that increase the overall effectiveness and reduce pharmaceutical adverse effects. A previous systematic review suggests, however, that the results should be interpreted cautiously, owing to methodological flaws of reported studies [24]. A rigorously designed randomized controlled trial to investigate the add-on effects of combining oral Chinese herbal medicine with conventional pharmacotherapy for the treatment of psoriasis vulgaris is warranted.

### Chinese herbal medicine formula development

The composition of the Chinese herbal medicine formula was determined based on previous clinical use, Chinese herbal medicine expert consensus and identified compounds of the ingredients that have antipsoriatic related pathways (such as anti-inflammation and antiproliferation) [25, 26]. Similarly, the botanical components have multiple Chinese herbal medicine functions that are likely to act on the known psoriasis Chinese medicine syndromes of blood heat, blood dryness or blood stasis. *Radix Paeoniae Rubra* (chi shao) has Chinese medicine functions of clearing heat and cooling blood whilst one of its key compounds, paeoniflorin, has shown to be a platelet aggregation inhibitor [27]. *Sarcandra glabra* (jiu jie cha) has the Chinese medicine function of reducing heat in the blood and contains the compound fumaric acid, which is a known longstanding treatment for psoriasis [28]. *Rhizoma Smilacis Glabrae* (tu fu ling) has the Chinese medicine function of eliminating toxic heat and syringic acid, one of its components, shows evidence of antibacterial action [29]. Other botanicals contained in YXBCM01 have the Chinese medicine functions of invigorating blood circulation, harmonizing other botanicals, tonifying *Qi* and removing heat from the blood. Further potential biological actions of the key components include: antineoplastic, immunosuppressive and enhanced anti-inflammatory and antibacterial activity.

## Methods/design

The study will investigate Chinese herbal medicine YXBCM01 combined with conventional therapy (calcipotriol) for psoriasis vulgaris in an Australian population with PASI severity ≥7 and ≤12. The study was developed in collaboration with a Chinese study also currently investigating YXBCM01 but combined with a different conventional therapy (calcipotriol betamethasone), for relapse rate in a Chinese population with psoriasis vulgaris of greater severity [30]. The Australian study is designed as a double-blind, randomized placebo controlled trial to determine the feasibility of the trial protocol, prior to undergoing a full scale trial. It will also provide indication of the potential efficacy and safety of combined YXBCM01 and calcipotriol, compared with calcipotriol and placebo. The trial protocol was approved by the RMIT University Human Research Ethics Committee, filed with the Therapeutic Goods Administration under the Clinical Trial Notification scheme and registered with the Australian and New Zealand Clinical Trials Registry, ACTRN12614000493640.

### Trial procedure

**Figure 1:**
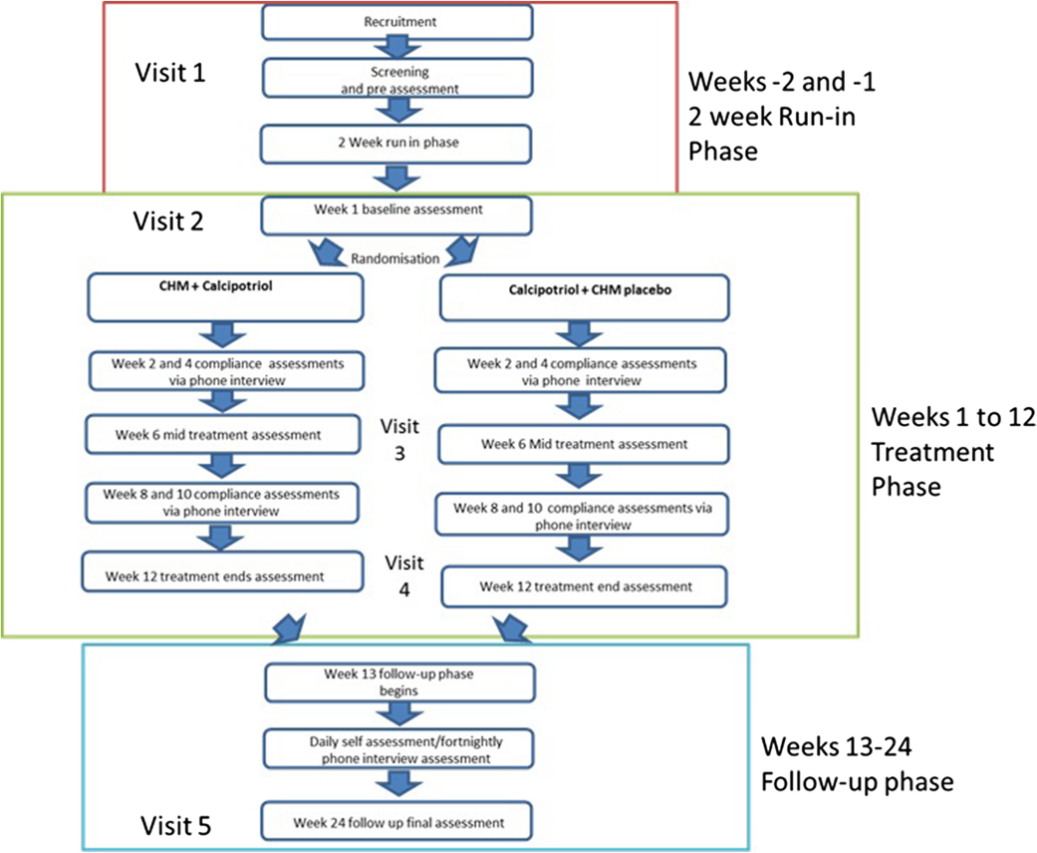
**Trial procedure flowchart.** CHM: Chinese herbal medicine.

The entire trial consists of an initial assessment, a two-week run-in phase (week -2 and -1), a 12-week treatment phase (week 1 to week 12) and a 12-week follow-up phase (week 13 to week 24) (Figure 1).

Potential participants will be invited to an initial assessment (first visit) consisting of inclusion and exclusion criteria screening. If eligible, participants will be asked for informed consent and undergo assessment of PASI [31], body surface area [32], Dermatology Life Quality Index [33] and Skindex 29 [34], as well as full blood count, kidney and liver function blood tests. Participants will then partake in a two-week run-in phase (weeks -2 and -1). During this period, psoriasis-related medication and herbal supplement use will be restricted. Participants will be provided with a moisturizer (sorbolene cream), to be applied as required during this period, to ease symptom discomfort until the treatment phase commences. After the run-in period, participants will undergo baseline assessments (second visit), including assessment of PASI, body surface area, Dermatology Life Quality Index, Skindex 29, and Chinese medicine theory of syndrome diagnosis (blood heat, blood dryness or blood stasis) [35]. Photographs of a typical lesion area will be taken, masking the identity of the participants; these will then be coded and placed in the participants’ case report forms [28].

After baseline assessments, participants will be randomized to receive 12 weeks of YXBCM01 plus calcipotriol, or placebo YXBCM01 plus calcipotriol treatment. During the treatment phase, psoriasis symptom measures will be self-reported by participants and recorded in a daily or weekly diary. Face-to-face assessments with blinded assessors will be scheduled at weeks 6 (third visit) and 12 (fourth visit), with further data collected including symptom severity, safety, trial drug usage, physiological tests and lesion photos (Table 1). Phone calls will be made to participants at weeks 2, 4, 8 and 10 to check adherence to the dosage guidelines and monitor adverse events.

**Table 1:** **Assessment measures and time points**

	PASI	Body surface area	Dermatology Life Quality Index	Skindex 29	Body mass index	Chinese medicine syndrome diagnosis	Medication and health utilization	Blood tests	Check adherence	Adverse events of Chinese herbal medicine and calcipotriol
Pre-assessment (Visit 1, Week -2)	×	×	×	×	×			×		
Baseline assessment (Visit 2, Week 1)	×	×	×	×	×	×				
Daily or weekly self-assessment during 8 weeks treatment (Weeks 1 to 12)							×			×
Compliance assessment phone call (Weeks 2 and 4)									×	×
Mid-treatment assessment (Visit 3, Week 6)	×	×	×	×	×			× (liver and kidney function only)	×	×
Compliance assessment phone call (Week 8 and 10)									×	×
End of treatment assessment (Visit 4, Week 12)	×	×	×	×	×	×	×	×	×	×
Daily or weekly self-assessment during 12 weeks follow-up (weeks 13 to 24)							×			×
End of follow-up assessment (Visit 5 or Week 24)	×	×	×	×	×	×	×	×	×	×

*PASI* Psoriasis Area and Severity Index.

Participants will record any reductions in calcipotriol dosage along with reasoning, in a daily diary. The YXBCM01 therapy and placebo initial dosages will be continued till the end of the 12-week treatment phase, regardless of psoriatic lesion clearance. Any unused trial medication will be collected at the end of treatment assessment and measured to assess compliance.

For the 12-week follow-up phase, participants will continue to record symptoms in their daily or weekly diaries and document their utilization of health resources (including drug therapy use). At week 24 participants, will attend an end of follow-up assessment (fifth visit); following this, the participants’ involvement in the trial will cease.

### Setting and participants

The study will be undertaken at the clinical trial facilities of the Research Hub of the School of Health Sciences, RMIT University, in Melbourne, Australia. Recruitment will be through poster advertisement, newspaper advertisement, newsletters and Internet advertisement. Participants might also be invited to contact trial coordinators by their treating physician (general practitioner, dermatologist or immunologist). Thirty participants will be included in the study after providing written informed consent. The selection criteria are as follows.

#### Inclusion criteria

Participants will be included only if they satisfy the following: (1) aged between 18 and 70 years; (2) at least a 12-month history of psoriasis vulgaris symptoms diagnosed by a physician and where calcipotriol would be appropriate treatment; (3) PASI score ≥7 and ≤12; (4) informed consent is provided.

#### Exclusion criteria

People with any of the following will be excluded from participation: (1) pregnant or breast-feeding; (2) psoriasis type is not vulgaris; (3) taking systemic drugs or phototherapy for psoriasis within 4 weeks prior to screening; (4) taking topical drug treatment for psoriasis within 2 weeks prior to screening; (5) other severe disorders; (6) known disorders of calcium metabolism (high blood calcium levels); (7) known kidney function disorders; (8) taking calcium, vitamin D supplements or vitamin D-like medicines; (9) known sensitivity to Chinese herbs; (10) known sensitivity to calcipotriol; (11) unwilling or unable to cease other topical or systemic psoriasis-related medication for duration of the trial.

### Interventions

#### Chinese herbal medicine

The oral Chinese herbal medicine granule (named YXBCM01) will be produced by a manufacturer holding a Good Manufacturing Practice certificate.

Developed by a renowned Chinese herbal medicine clinician (Professor Guowei Xuan), YXBCM01 includes *Radix Paeoniae Rubra* (chi shao), *Sarcandra glabra* (jiu jie cha), *Rhizoma Smilacis Glabrae* (tu fu ling). A previous observational study of YXBCM01 for psoriasis vulgaris showed a reduction in PASI and Dermatology Life Quality Index score with no significant adverse events [36]. The formulation is registered with the Guangdong Food and Drug Administration (hospital preparation approval number Z20080123). The botanicals will be mixed, cooked, filtered and pressure spray-dried, forming granules. These will be packaged in small single-dose sachets (9.5 cm × 6.0 cm), weighing 5.5 g each. The intervention group will receive individually packaged doses (*n* = 168) of YXBCM01, with each dose to be dissolved in warm water and consumed orally twice daily for 12 weeks.

#### Placebo

Placebo will be produced by the same manufacturer as the YXBCM01 granules and consist of starch with no active ingredients. It will be matched as closely as possible to the appearance and taste of the YXBCM01 granules. The colour will be made identical by adding artificial pigment whilst the taste will be adjusted by adding a medicine intermediate.

Dosage and administration instructions for the YXBCM01 and placebo groups will be identical.

#### Pharmacotherapy

The topical drug calcipotriol 0.005% (50 μg/g) cream (30 g tubes) will be administered as standard care. Calcipotriol is a vitamin D3 derivative, which decreases proliferation and induces the differentiation of keratinocytes. It has a strong immunomodulating effect, reducing the level of pro-inflammatory cytokines [37, 38]. It is a moderate-action first-line therapy drug, with a low risk of side effects and proven efficacy; it is commonly used and treatment guidelines recommend it for mild to moderate psoriasis [39]. The calcipotriol cream will be administered daily, for 12 weeks, to affected body surface areas according to American Academy of Dermatology guidelines (1% surface area coverage = 0.5 fingertip units), or until the complete clearance of lesions [15]. The maximum dose will not exceed 100 g per week, according to consumer medicine information for the product (calcipotriol). The calcipotriol dosage can be reduced from its initial dose at the participants’ discretion and as their symptoms reduce, but should meet dosage recommendations for the severity of the plaques. In addition, in case of intolerable itch, antihistamine (cetirizine hydrochloride tablets 10 mg) will be provided to all participant groups as rescue therapy.

### Objectives

The objectives of this randomized controlled trial are:

- To investigate any add-on benefit of oral Chinese herbal medicine (YXBCM01) to calcipotriol for the management of psoriasis vulgaris;
- To investigate whether combination of oral Chinese herbal medicine (YXBCM01) and calcipotriol is safe for the management of psoriasis vulgaris.

### Outcome measures

#### Primary outcome measures

The primary outcome measure will be PASI change (% mean) from baseline to end of treatment (week 12) and end of follow-up phase (week 24) (see Table 1).

#### Secondary outcome measures

- PASI 75 (proportion (%) of participants achieving a PASI improvement of 75%) (week 12);
- PASI 50 (proportion (%) of participants achieving a PASI improvement of 50%) (week 12);
- Relapse rate (return of lesion to 50% of baseline PASI score [40]) (week 24);
- Change in body surface area (%) (week 12);
- Change in Dermatology Life Quality Index score (%) (week 12);
- Skindex 29 score change (%) (week 12);
- Acceptability and willingness to repeat treatment (week 12);
- Blood test (full blood, kidney and liver function tests) (weeks 6, 12 and 24);
- Body mass index change (%) (week 12);
- Reported adverse events and serious adverse events;
- Health resource utilization data (medical doctor visits, hospital visits and medication usage).

Acceptability will be measured on an ordinal scale from 0 to 10, where 0 equals very dissatisfied with the treatment and 10 equals very satisfied with the treatment. Willingness to repeat the treatment will be measured on a Likert scale (definitely not, probably not, probably yes, definitely yes or unsure) (week 12). Any adverse events will be recorded by the participant and assessed by blinded assessors at each assessment period.

A full blood count test, as well as liver and kidney function tests, will be completed at weeks -2, 6, 12 and 24 (Table 1). These will measure the impact of the interventions on blood inflammatory markers, as well as monitoring toxicology safety for significant changes in kidney and liver function. Samples of serum will be collected and stored for further investigation of changes to the concentration of key cytokines (weeks -2, 12 and 24). Adverse events will be recorded from participants’ reports and assessors’ observations, then classified as probably related to either Chinese herbal medicine or pharmacotherapy. Any serious adverse event will be reported to RMIT University Human Research Ethics Committee, a data safety and monitoring board and the Therapeutic Goods Administration of Australia. Health resource utilization data will be collected in case report forms; data will include number and reason of hospitalizations, number of medical doctor related visits, reason for visit and use of medication related to psoriasis (frequency and dosage).

### Potential adverse events

Potential adverse events of calcipotriol are considered minor and may consist of minor skin irritation (including peeling or rash), change in skin colour or skin sensitivity to light [41]. For the YXBCM01, the herbal constituents are commonly used clinically. Minor side effects of YXBCM01 are possible but are not specific to the investigated herbal substances; although uncommon, they may consist of nausea or diarrhoea [42]. If participants develop adverse events or if the results of their blood tests for liver and kidney function become abnormal, participants will be advised to stop all trial-related medication and seek medical advice from their doctors as to their safe continuation in the study.

### Termination and withdrawal

A data safety and monitoring board of independent experts in pharmacological studies, dermatology and statistics has been formed. The data safety and monitoring board will review and evaluate the study data for safety and study conduct, to make recommendations regarding the continuation, modification or termination of the study. Serious adverse events will be judged according to Therapeutic Goods Administration criteria (2006): ‘Any untoward medical occurrence that at any dose: results in death, is life-threatening, requires inpatient hospitalization or prolongation of existing hospitalization, results in persistent or significant disability/incapacity, is a congenital anomaly or birth defect, or is a medically important event or reaction’ [43]. If judgement is made by the relevant human research ethics committee, Therapeutic Goods Administration, data safety and monitoring board or study investigators that participants are at serious risk as a result of serious adverse event reported in the study, the study will be terminated. In addition, participants are permitted to withdraw at any time throughout the duration of the trial, with or without reason. All withdrawn participants who have received the intervention will be followed-up 8 weeks post withdrawal, to obtain information regarding their condition and any subsequent adverse events.

### Sample size determination

A pilot study of 30 participants testing the feasibility of the trial protocol will be conducted prior to a full scale trial. Post-hoc power analysis via effect size estimation on the pilot study data will be used to determine sample size for a full scale trial.

### Randomization, sequence generation and allocation concealment

Participants will be randomly allocated to intervention (YXBCM01 plus calcipotriol) or control group (placebo plus calcipotriol) in equal ratio (1:1). Randomization numbers will be computer generated in blocks and corresponding codes sealed in opaque envelopes. Envelopes will be opened sequentially from each block only after the participant’s details are written on the outside of the envelope. The code inside the envelope will correspond to a package number that will contain 12 weeks supply of either YXBCM01 granules or identically packaged placebo granules.

### Blinding

Participants, researchers and outcome assessors will be blind to group allocation. Packaging and labelling of study drugs will be conducted by persons independent of the research team. The randomization sequence will be sealed and stored in a locked filing cabinet, separate from participants’ data and only accessible to persons who generated the sequence. Testing of adequate participant blinding will be undertaken at weeks 6, 12 and 24; participants will be asked whether they believe they were given placebo, Chinese herbal medicine or are unsure. Randomization codes will only be broken after data validation and entering processes are complete, or at the request of the human research ethics committee, Therapeutic Goods Administration or the participant’s own physician, if there is identified risk to a participant’s continuation in the study.

### Data collection

Data will be collected and recorded in the case report forms from participants via face-to-face and telephone interviews or assessments. Participants will record further daily and weekly data in a home data diary, to be collected by researchers at subsequent assessment or review appointments.

All data will be entered into a pre-designed, password-protected dataset by personnel blinded to group allocation. Data entry will be performed continuously throughout the study using the double-check method, with any correction or changes of written data in participants’ case report forms documented and dated.

### Statistical analysis methods

All data will be analyzed by an independent statistician. The Statistical Package for the Social Sciences software version 21.0 for Windows (SPSS Inc., Armonk, New York, USA) will be used for data analysis. Baseline demographic characteristics on categorical variables, such as sex, will be assessed for balance between the two treatment groups via the chi square test, whilst continuous or interval variables will be assessed for equivalence between the treatment groups by *t* tests. Intention-to-treat analysis will be applied to outcome data to minimize bias due to withdrawals; all missing data will be replaced using the last observation carried forward. Continuous data will be presented as means and standard deviation, or 95% confidence interval. All dichotomous data, such as the percentage of participants achieving PASI 75, will be presented as risk ratio and 95% confidence interval. Other outcomes (for example, severity and quality of life) will be assessed for equivalence in the two groups; *P* < 0.05 obtained from statistical tests on variables involving treatment group comparisons will be considered statistically significant.

## Discussion

### Outcome measures used in this study

From research, complete clearance of psoriasis symptoms is rarely achievable; as a result, treatment success is based on reduction in PASI of 75% (PASI 75) [44]. Treatment failure is regarded as a reduction in PASI of less than 50% (PASI 50). For those PASI changes falling between this range (≥50% but <75%), the Dermatology Life Quality Index is used to determine whether current treatment should be modified or continue [45]. The Therapeutic Goods Administration recommends standard outcomes of PASI 75, PASI 50 and body surface area to assess product efficacy for psoriasis [44]. The Dermatology Life Quality Index is recommended by both the Therapeutic Goods Administration and the Australian College of Dermatologists, to evaluate psoriasis severity and patients’ quality of life [12, 44]. It is a validated instrument that is well-recognized in psoriasis research. The Skindex 29 has been evidenced for psoriasis as the most sensitive scale, with high applicability; previous evaluation identifies it as the most validated quality-of-life instrument for dermatological conditions [46, 47].

### Chinese medicine syndrome approach

As yet, there is no conclusive evidence that utilizing Chinese medicine syndrome theory in clinical trial treatment will improve the efficacy of treatment [48]. Whilst this study will not allocate treatment based on syndrome differentiation, it will be able to analyze the results of the intervention and investigate whether there are differences in efficacy between participants in each syndrome group (blood heat, blood dryness or blood stasis). With text books differing, there is conjecture about the number of Chinese medicine syndrome types for psoriasis; the development of the Chinese medicine syndrome differentiation criteria for this study was based on available Chinese guidelines, as well as expert consensus [23].

## Trial status

The trial is currently recruiting, following the protocol stipulated in the Australian and New Zealand Clinical Trials Registry.

## Abbreviations

PASI: Psoriasis Area Severity Index
PASI 50: reduction in PASI of less than 50%
PASI 75: reduction in PASI of 75%.
